# Mapping the Landscape of Environmental Health Literacy: Trends, Gaps, and Future Directions

**DOI:** 10.3390/ijerph23020140

**Published:** 2026-01-23

**Authors:** Bernardo Oliveira Buta, Marjorie Camila Madoz Pinheiro, Benjamin Miranda Tabak

**Affiliations:** School of Public Policy, Government, and Business, Getulio Vargas Foundation (FGV EPPGE), Brasilia 70830-020, Brazil; marjorie.unb@hotmail.com (M.C.M.P.); benjamin.tabak@fgv.br (B.M.T.)

**Keywords:** environmental health literacy, health literacy, public health, climate change, bibliometrics, review

## Abstract

Environmental Health Literacy (EHL) empowers individuals and communities to understand and make informed decisions about health and the environment. This study uses bibliometric indicators to map the field, identifying patterns, emerging trends, and gaps that offer opportunities for future research. We analyze 152 articles from PubMed, Scopus, and Web of Science databases. The first publication was recorded in 2012, and there was a significant increase in output since 2018. The literature emphasizes environmental exposures and public health, with a growing focus on social justice and participatory research. While areas such as environmental exposure, environmental health, health literacy, and participatory research are well established, significant gaps remain in emerging and cross-cutting themes, including education, health risks, environmental/climate justice, community engagement, communication, and climate-related health literacy. These issues are increasingly central to debates on the intersection of health, environment, and social equity, as they are key to advancing environmental justice, reducing health inequalities, and empowering vulnerable populations to make informed decisions, contributing to the development of more inclusive and effective public policies.

## 1. Introduction

In recent decades, Environmental Health Literacy (EHL) has established itself as a key multidisciplinary field for understanding and mitigating the impacts of environmental exposures on human health. Initially defined as the ability to recognize the connection between environmental exposures and health [1], EHL has expanded to include a set of competencies that empower individuals and communities to make informed decisions with the aim of reducing risks and improving their living conditions [2,3]. By integrating areas such as public health, environmental sciences, and risk communication, EHL stands out as a comprehensive concept, anchored in preexisting literacies such as scientific and health literacy [1].

Environmental Health is a branch of public health focused on the relationship between environmental exposures and human health. In other words, this field examines how the environment impacts people’s health, covering health problems caused by environmental factors of various kinds, whether physical, chemical, or biological [1,4]. By focusing specifically on the interface between human health and the environment, this field differs from other fields of knowledge, such as environmental science and environmental engineering.

The low level of literacy among individuals and communities regarding the health risks of environmental exposure can have significant impacts. In Rhode Island, for instance, residents who do not understand the risks of consuming fish contaminated with mercury tend not to change their eating habits [5]. Owners of private wells in the United States consume water contaminated with toxic materials that are odorless or invisible to the naked eye, believing that the water is safe because it tastes, smells, and looks good [6]. Residents of Suriname tend not to take safety measures against the proliferation of disease vectors, such as the dengue mosquito, because they do not fully understand how arboviruses are transmitted [7].

The promotion of EHL has proven particularly effective in vulnerable communities that are exposed to significant environmental contaminants, such as oil refinery pollutants and heavy metals in water sources, which generate serious health problems [8,9]. The practice of sharing the results of environmental exposure studies, as exemplified by Brody et al. (2014a) [8], strengthens awareness of the risks faced by participants and promotes collective actions to reduce these exposures, as well as driving demands for more effective public policies.

In addition, the analysis of cumulative impacts and risk assessments in marginalized communities reveals significant insights into how EHL can be applied to address environmental disparities. Exposure studies in environmental justice communities go beyond simple data collection, promoting a more comprehensive understanding of the social and economic factors that influence exposure patterns [8,10]. These investigations reinforce the need for a collaborative and integrative approach that involves communities in developing sustainable and culturally appropriate solutions to improve environmental health.

This article shows that the literature on EHL has gained greater relevance over time and has transitioned from a more theoretical approach to a practice-oriented approach focused on health interventions that support public policies. There is an important interface between the literature on environmental health literacy and citizen science. Report-back practices and the active involvement of the community in diagnosing environmental health risks and mitigating problems tend to increase self-efficacy and facilitate the adaptation of health behaviors. Citizen science addresses issues of equity by giving a voice to disadvantaged communities [11,12,13,14]. This is key to solving complex public problems. In fact, involving the community in the search for solutions and acting in cooperative networks tends to generate more effective and legitimate public policies [15].

Given the growing relevance of EHL as an emerging area and the need for studies that can systematize existing knowledge and map the main characteristics of academic production, the purpose of this study is to map the evolution of EHL to identify how scientific evidence can support the formulation of more effective public health policies. This study describes the scientific production on EHL through bibliometric indicators, identifying patterns, emerging trends, and gaps that offer opportunities for future research and development of the field. This contribution is methodologically sophisticated, as bibliometric studies involve literature reviews conducted through quantitative analysis based on the statistical measurement of scientific activity. While numerous software programs are available for such analyses, this study used the R statistical software (version 2023.12.1+402) and its packages: bibliometrix, dplyr, tidyr, and ggplot2. The Bibliometrix package offers not only statistical tools but also a comprehensive workflow for scientific mapping [16].

## 2. Methods

### 2.1. Data Sources and Extraction

The data for this study were extracted from the PubMed, Scopus, and Web of Science databases with the aim of carrying out a bibliometric analysis on the subject of Environmental Health Literacy. The search query was “Environmental Health Literacy”. The search was originally conducted on 3 June 2024, and updated on 7 July 2025. Only academic articles and reviews published in English were considered. Publications in other languages or those that did not correspond to the specified types of documents were excluded. The specific searches used in each database are described below:

- Pubmed: (“environmental health literacy”[Title/Abstract]) AND (english[Filter])
- Scopus: TITLE-ABS-KEY (“environmental health literacy”) AND (LIMIT-TO (DOCTYPE, “ar”) OR LIMIT-TO (DOCTYPE, “re”)) AND (LIMIT-TO (LANGUAGE, “English”))
- Web of Science: (TS=(“environmental health literacy”)) AND (DT==(“ARTICLE” OR “REVIEW”) AND LA==(“ENGLISH”))

### 2.2. Selecting Records

The initial search identified 432 articles. Specifically, the PubMed database generated 122 articles, Scopus returned 171 articles, and Web of Science listed 139 articles. After applying a screening based on document type, the eligible articles resulted in a total of 284. Ten of these articles were excluded for not being in English. The 274 articles were consolidated into a single set using R, and 122 duplicate articles were identified and removed, resulting in 152 articles, ensuring that the subsequent analysis was carried out on a coherent and relevant data set.

**Figure 1:**
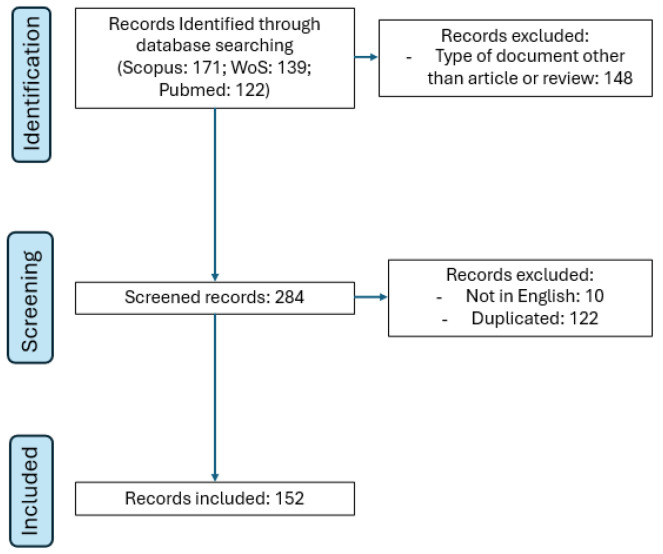
Flow diagram for the literature review.

A flowchart adapted from PRISMA (Preferred Reporting Items for Systematic Reviews and Meta-Analyses) was drawn up to illustrate the process of selecting the articles included in the study. PRISMA is a minimum set of evidence-based guidelines designed to improve transparency and accuracy in the presentation of systematic reviews and meta-analyses [17]. As highlighted in Figure 1, the PRISMA application facilitates the clear and objective organization of the study selection process.

### 2.3. Data Visualization, Interpretation and Analysis

To understand the development and impact of the field over time, we focus on analyzing the main metrics of scientific production, which provide a clear view of the evolution of publications, the impact of citations, and collaboration among authors. This allows for the identification of trends, growth patterns, and the academic relevance of the area studied.

Therefore, several variables were considered for the bibliometric analysis, such as the production of the most prolific authors over time, direct citation networks, growth of sources, impact of journals measured by the H-index, most cited articles, prominent affiliations, leading countries in scientific production, most frequent keywords, co-citations of authors and documents, coupling clusters, and the evolution of keywords.

The analysis was conducted with RStudio (version 2024.04.2), using the “Bibliometrix 4.1.0” package to evaluate the interrelationships of the components analyzed, as well as the dplyr, tidyr, and ggplot2 packages. The bibliometrix package enabled the creation of quantitative calculations and detailed visualizations, ensuring a systematic, transparent, and reproducible study in line with the recommendations of the scientific literature [16]. Table 1 presents the bibliometric indicators used in this research and their purposes.

## 3. Results

Based on the results presented, it can be seen that the EHL dataset spans the period from 2012 to 2025, totaling 152 documents published in 84 different sources. The annual growth rate of publications is 16.15%. The average age of 3.93 years per document indicates the average time elapsed since a document’s publication date until the present. The average number of citations per document is 11.26, and the total number of references cited is 5314. International collaboration is present in 11.84% of the documents. Regarding the types of publications, most of the documents are empirical articles (143), with a small presence of reviews (9).

### 3.1. Annual Scientific Production

**Figure 2:**
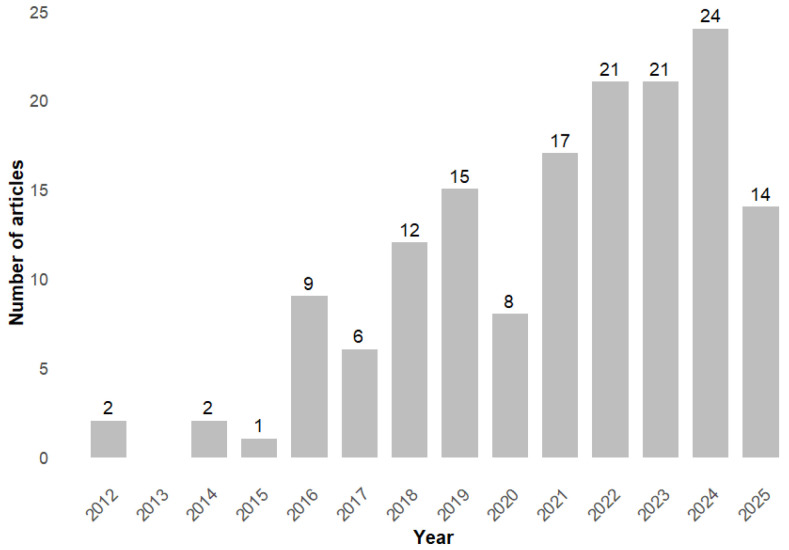
Number of articles published per year.

Figure 2 shows that 2012 was a milestone in publications on the subject, with a modest start in scientific production. Between 2012 and 2017, the number of articles published remained below 10 per year, and there was no production on the subject in 2013. As of 2018, there has been a significant increase in the volume of publications, culminating in a peak in 2024, with 24 articles published. This increase indicates a growing recognition of the relevance of EHL. By 7 July 2025, 14 publications had already been registered. This reinforces the continued importance and growing interest of the academic community in the subject.

#### Average Annual Citations per Article

**Figure 3:**
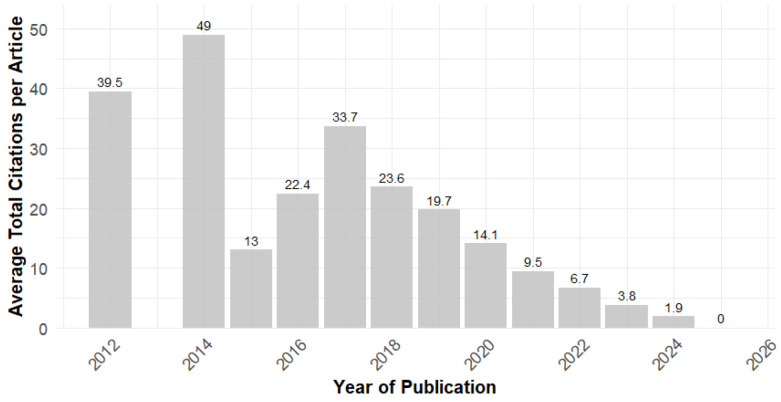
Average Total Citations per Article.

Figure 3 shows the evolution of average annual citations per article between 2012 and 2025. It was calculated for each year by summing the total citations of all articles published in that year and then dividing by the number of articles published in the same year. It is possible to observe a significant peak in 2014, which is a year with few publications that have made a large impact, considerably raising the average. After 2014, there was a sharp drop in 2015, followed by a recovery between 2016 and 2017, and a gradual decline from 2018 onward. As shown in the upcoming Figure 8, the most cited article is from 2017 [1]. This oscillation may reflect the fact that EHL is a relatively new and still developing field.

### 3.2. Most Relevant Sources

**Figure 4:**
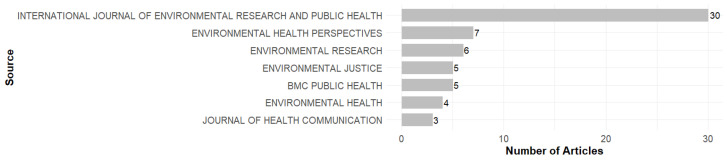
Most Relevant Sources.

Figure 4 shows the most relevant journals in terms of publications. The International Journal of Environmental Research and Public Health stands out as the most prolific journal, with 30 articles published. Environmental Health Perspectives published 7 articles. Other journals, such as BMC Public Health, Environmental Justice, Environmental Research, Environmental Health, and the Journal of Health Communication, had a moderate output, with publications ranging from 3 to 6 documents.

The journals Environmental Health Perspectives and Environmental Justice were pioneers in the production of articles related to EHL, particularly the articles by Brown et al. (2012) [10] and Ramos et al. (2012) [18], respectively. From 2016, the International Journal of Environmental Research and Public Health has begun to publish more consistently on the subject, with the article by Ramirez-Andreotta et al. (2016a) [2], and despite its late start compared to other journals, it quickly consolidated itself as the most prolific, especially from 2021. Environmental Research followed this trend in 2017, with the article by Ohayon et al. (2017a) [19] shows its involvement with the subject. In 2018, BMC Public Health also stood out with the publication of the article by Cláudio et al. (2018a) [20].

Table 2 presents an evaluation of the impact of journal publishing on EHL, based on the H, G, and M indices. The H-index reflects the balance between productivity (number of publications) and the impact of citations, while the G-index measures the distribution of citations among articles. In turn, the M-index makes it possible to compare the impact over time [21].

In general, the International Journal of Environmental Research and Public Health stands out as the most influential journal, with an H-index of 13, a G-index of 21, and an M-index of 1.3. Other journals, such as Environmental Health Perspectives, Environmental Research, BMC Public Health, and Environmental Justice, also stand out for their relevant contributions to the field.

### 3.3. Most Prolific Authors

**Figure 5:**
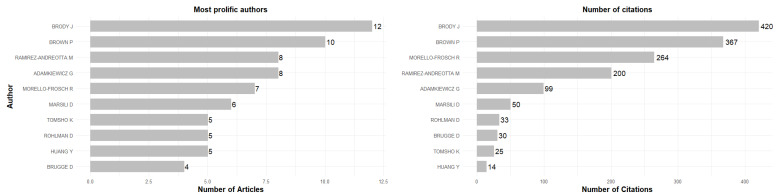
The most prolific authors.

The most relevant authors in terms of the number of publications in the field of EHL include Brody J., who leads with 12 published articles, followed by Brown P., with 10 publications. Ramirez-Andreotta M. and Adamkiewicz G. have 8 articles each. Morello-Frosch R. and Marsili D. have 6 and 5 publications, respectively. These authors represent the main contributors to the development of EHL research. This productivity data is shown in Figure 5, while their specific impact and research consistency are detailed in the following subsections.

#### 3.3.1. Production over Time

**Figure 6:**
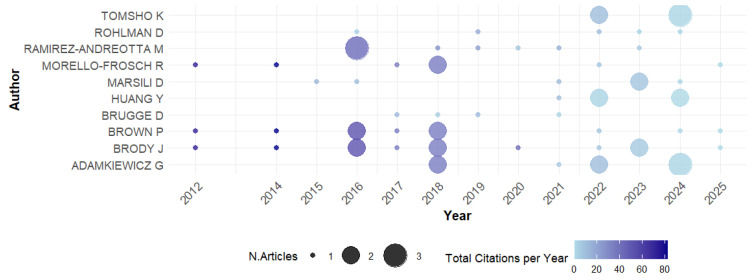
Author’s production over time.

An analysis of the authors’ output over time reveals different patterns of consistency and involvement with the field of EHL. Figure 6 shows the academic output over time of the most prolific authors. Brody J. shows the most consistent production, with regular publications from 2012 to 2023, standing out as one of the most prolific and active authors throughout the period analyzed. Brown P. also maintains a constant production, starting in 2012 and continuing until 2022, although there has been a slight reduction in the frequency of publications in recent years. Ramirez-Andreotta M. shows a pattern of high activity from 2016, with significant peaks in production between 2016 and 2018, and remains active until 2023. Marsili D. and Comba P. began publishing in 2015 but have not maintained the same regularity over the years, resulting in sporadic production in subsequent years. Adamkiewicz G. started publishing more recently, in 2018, and has shown a progressive increase in publications.

#### 3.3.2. Lotka’s Law

Lotka’s Law suggests that a small number of authors are responsible for the majority of publications, while the majority contribute only one or two articles. This comparison makes it possible to assess the degree of consistency between the authors’ actual productivity and the theoretical prediction, revealing the authors’ performance in the field studied [22]. The data show that empirical production is close to theoretical expectations, with the majority of authors (82.4%) publishing just one article. In comparison, only 11.2% of the authors published two articles, and 6.2% published three articles or more.

#### 3.3.3. Impact Measurements

Table 3 shows the most influential authors in the field of EHL based on the H, G, and M indices. Brody J. stands out as the author with the greatest impact, leading with an H-index of 9, a G-index of 12, and having 420 total citations in 12 articles since the start of his publication in 2012. Brown P. ranks second, with an H-index of 7, a G-index of 10, and 367 citations in 10 articles, beginning in 2012. Ramirez-Andreotta M. follows with an H-index of 7, a G-index of 8, and an M-index of 0.7, consolidating her position as one of the leading researchers in the field, with 200 citations accumulated since 2016. Morello-Frosch R. is also among the leading authors, with an H-index of 6, a G-index of 7, and accumulating 264 citations in 7 publications since 2012. Among the most recent authors, Adamkiewicz G. began publishing in 2018 and has an H-index of 5, a G-index of 8, and 99 citations in 8 publications to date, standing out for his rapid rise in the field.

### 3.4. Affiliations

**Figure 7:**
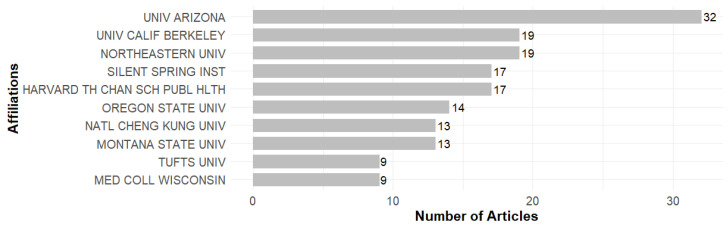
Top 10 affiliations.

The authors who publish in EHL are affiliated with institutions that play a key role in advancing and disseminating knowledge in the field. An analysis of the most relevant affiliations, presented in Figure 7, reveals that the University of Arizona stands out as the most productive institution, with 32 publications, leading the way in terms of academic contributions. Other prominent institutions include the University of California, Berkeley, and Northeastern University, with 19 publications each.

Several US institutions appear in the top ten, reflecting the prominence of the United States in the production of research on EHL, as shown in Figure 7. This shows that, although there are contributions from institutions in other countries, such as Italy and Taiwan, US institutions dominate both in terms of volume and impact.

#### Scientific Production by Country

The number of articles published indicates productivity in a specific field. The number of citations indicates the impact of these publications. Table 4 highlights the most prolific countries in terms of the number of scientific publications and citations. The basis for counting considers the authors’ affiliations, so articles can be counted more than once because they have more than one author. In other words, multiple counting occurs when there is co-authorship among multiple countries.

The United States occupies a prominent position, with a total of 441 productions and 1442 citations, followed by China, with 50 productions and 41 citations, and Italy, with 35 productions and 53 citations. Other notable countries include Australia, Thailand, the United Kingdom, and Canada, with 24, 14, 14, and 10 productions, respectively. This distribution highlights the leadership of the USA in knowledge production on the subject but also the growing contributions of other countries to the development of the field.

### 3.5. Most Cited Articles

**Figure 8:**
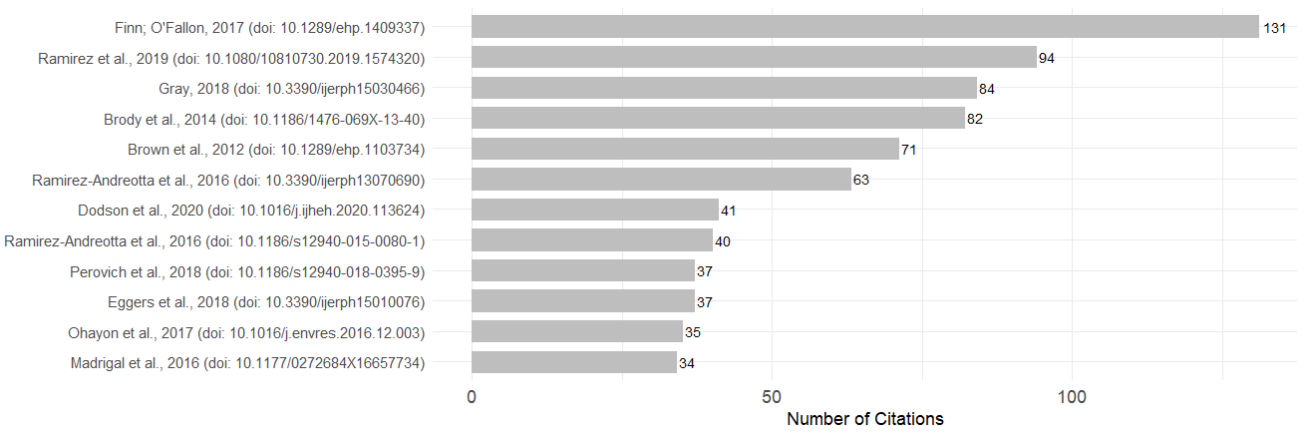
Most frequently cited articles [1,2,3,8,9,10,19,23,24,25,26,27].

The most cited articles in the field of EHL offer a comprehensive overview of the main theoretical contributions, trends, practices, and emerging topics. Table 5 summarizes the key characteristics and findings of these seminal works, and Figure 8 presents the most frequently cited articles.

**Table 5:** Summary of the most cited articles and their key contributions to EHL.

Reference	Key Contributions
Finn & O’Fallon (2017) [1]	**Presents a definition and conceptual model for EHL, placing it in its social and historical context.** That work also identifies complementary areas and domains in which EHL is being applied, and outlines a research agenda for the future. The authors state that EHL can potentially benefit the conduct and outcomes of community-oriented environmental science and health disparities research by ensuring that the translation of scientific findings to people results in a greater understanding of specific risks, reduced exposures, and improved health outcomes for individuals and communities.
Ramírez et al. (2019) [23]	**Examine the challenges and opportunities in air pollution communication, highlighting gaps in clarity and responsibility for communication, insufficient information on risk mitigation, and long-term health impacts.** The study also highlights the disparities in the reach of information, particularly in relation to the most vulnerable populations. The authors emphasize the importance of a more inclusive and effective approach to EHL, with the aim of empowering communities to face environmental threats in a more proactive and informed manner.
Gray (2018a) [3]	**Offers an analysis of the representation and measurement of EHL in academic literature, highlighting the challenges involved in people understanding and use of information about environmental risks.** The author emphasizes the crucial role of EHL in empowering individuals and communities to act more effectively to protect public health, pointing to the need to develop more robust and varied evaluation methods. Based on the studies reviewed and the principles of community-based participatory research (CBPR), Gray (2018) [3] proposes that EHL can be more effective by promoting action at both the individual and community levels, with the potential to encourage health protection decisions in a more comprehensive and sustainable manner.
Brody et al. (2014) [8]	**Address the ethical and practical implications of sharing biological monitoring results with study participants.** The article emphasizes the importance of providing personalized feedback to participants, arguing that transparency in this process offers several benefits, such as increased trust in science, greater participation in long-term studies, improved EHL, individual and community empowerment, and greater motivation to reduce environmental exposures.
Brown et al. (2012) [10]	**Studies community-based participatory research (CBPR), highlighting its role in promoting environmental justice through partnerships between researchers and communities.** The article emphasizes the importance of evaluating the impact of research on environmental health literacy, not only based on scientific publications but also by considering the community benefits, social empowerment, and political changes generated.
Ramirez-Andreotta et al. (2016a) [2]	**Discuss the impact of communicating the results of environmental exposures on promoting social justice and empowering vulnerable communities.** The study highlights how exposure feedback can increase EHL, empower individuals to take action to reduce risks, and engage the community in collective action to tackle environmental issues. In addition, the research points out that transparent and accessible communication is essential for establishing trust between participants and researchers, promoting greater community participation in public health initiatives.
Dodson et al. (2020) [24]	**Investigates whether consumer behavior related to avoiding certain chemicals is associated with exposure to endocrine-disrupting chemicals.** Through biomonitoring of large numbers of people, the study concludes that individual behaviors aimed at avoiding the consumption of certain products tend to be effective in reducing exposure to specific substances. However, this effectiveness is limited by the lack of transparency in the labeling of products such as sunscreens, body lotions, face creams, etc.
Ramirez-Andreotta et al. (2016b) [25]	**Reinforces the importance of returning environmental exposure information to participants in order to raise awareness and promote EHL.** The study highlights how the return of personalized information can improve the understanding of environmental risks and empower individuals and communities to make informed decisions and take protective action. By involving participants in interpreting the results, the research fosters self-directed learning and encourages active engagement in environmental health issues.
Perovich et al. (2018) [26]	**Discuss the ethical and public health implications related to environmental exposure in low-income communities.** The practice of report-back on environmental exposures is seen as a good research practice and contributes to the promotion of environmental health literacy, given the population’s greater understanding of environmental health risks.
Eggers et al. (2018a) [9]	**Analyzes the cumulative risks of exposure to contaminants in indigenous communities in the USA, highlighting the importance of community participation in identifying and mitigating these risks.** The study reveals that the lack of knowledge about environmental safety exacerbates the vulnerability of these populations, reinforcing the urgent need to improve EHL.
Ohayon et al. (2017a) [19]	**Explores the return of biomonitoring data to participants in environmental exposure studies.** The article emphasizes the importance of this practice for increasing EHL and empowering individuals and communities to make decisions that reduce environmental risks. The benefits include greater confidence in science, behavioral changes to reduce exposure, and mobilization to influence public policy. There are also ethical and practical challenges, such as communicating results on contaminants for which there are no established health guidelines and the need to adapt feedback to vulnerable populations.
Madrigal et al. (2016a) [27]	**Uses participatory research to explore the reduction in chemical exposure in young Latinos.** The research involved young people collaborating on a project aimed at monitoring endocrine disruptors in cosmetics. In addition to promoting EHL, the participants developed leadership skills and community engagement. The study highlighted the effectiveness of educational initiatives and the importance of the active participation of young people in the research process, emphasizing how this approach can empower vulnerable communities to face challenges related to environmental health and promote behavioral changes.

#### 3.5.1. Most Relevant Words

**Figure 9:**
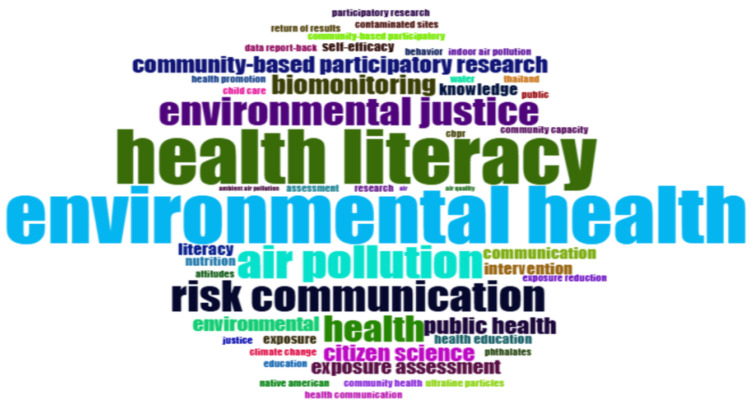
Most relevant words.

The keywords most commonly used in the publications demonstrate the relevance of these areas in the field of study. The word-cloud presented in Figure 9 clearly highlights the most commonly used terms in publications on EHL. As can be seen, the terms environmental health, health literacy, environmental justice, air pollution, and risk communication are very prominent, highlighting their central role in discussions on the subject. These three key terms act as conceptual anchors for contemporary research into EHL, reflecting the predominant focus on studies examining environmental exposures and their direct impact on human health.

In addition, relevant terms such as community-based participatory research, biomonitoring, citizen science, and exposure assessment also feature prominently, albeit less frequently. Although they represent important topics, they do not yet receive the same level of attention as the key terms. Although the term ’climate change’ is present, topics related to it and the effects of extreme weather events have not yet emerged as central or highly frequent themes in the current EHL literature, despite the increasing frequency of such events.

#### 3.5.2. Trend Topics

**Figure 10:**
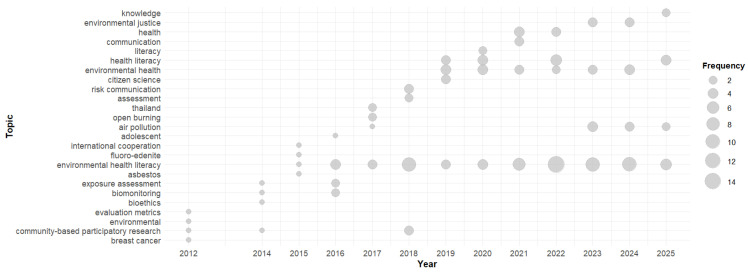
Temporal Evolution of the Main Topics.

The topic trend analysis shows the most frequent keywords over time, revealing the usage of certain words during different periods. As presented in Figure 10, there has been an expansion in the number of topics over the years. Topics such as Environmental Health Literacy, environmental health, health literacy, and air pollution consistently appear over the years, representing the conceptual pillars of this area of study. The most recently discussed topics include environmental justice, knowledge, and communication; they can be viewed as directions in this area of research.

### 3.6. Coupling Maps

**Figure 11:**
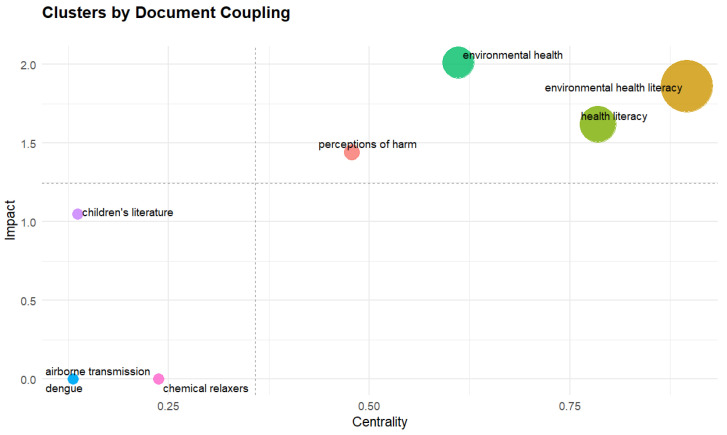
Coupling Maps.

The coupling map identifies groups of articles that share common references, suggesting the formation of subfields or research clusters. Figure 11 illustrates the prominent keywords emerging from the groups created through Clustering by Coupling. To carry out this process, the following parameters were applied: coupling by references, impact measure by global citation score, and labeling by authors’ keywords. In addition, the number of units used was 250, the minimum cluster frequency was set at 0 and 3 labels were used for each.

Four clusters were identified, organized in the graph based on two axes: impact, which reflects the degree of influence of each topic; and centrality, which indicates how much the topic is connected to other relevant topics. In the upper right quadrant, the clusters contain topics such as environmental health, environmental health literacy, health literacy, and perceptions of harm, which have high centrality and impact.

The clusters located in the lower left quadrant deal with more specific topics that are less directly connected to the central core of EHL: dengue, airborne transmission, chemical relaxers, and children’s literature.

### 3.7. K-Structures Framework

K-structures are used in bibliometric analysis to organize and categorize the interconnections between various elements of scientific production, such as authors, themes, and institutions. It is divided into three main categories: conceptual, intellectual, and social. This classification helps to understand the evolution and connections between knowledge and collaboration within a field of study. Applying this framework allows for a detailed analysis of scientific production patterns, revealing how knowledge is created, disseminated, and how researchers interact with each other [16].

#### 3.7.1. Conceptual Structure

The conceptual structure refers to the themes and concepts explored in the scientific literature, reflecting the content of the publications. Its analysis generally involves looking at the keywords and topics discussed in the articles [16]. The concepts are then analyzed using co-occurrence networks, thematic maps, and dendrograms.

The graphic presentation of networks of the most representative words is a visual technique that makes it possible to observe the relationships between terms and concepts surrounding a specific theme. This technique is based on the co-occurrence of words, mapping the frequency with which certain terms appear together in keywords, abstracts, titles, or even in the entire body of a set of publications. From these co-occurrences, it is possible to identify how certain concepts are connected, offering a clear view of the predominant and emerging thematic areas in a field of study [21]. Each cluster is represented by words that stand out for their frequency and importance, demonstrating the structure of knowledge around a topic and its links with other areas [21].

**Figure 12:**
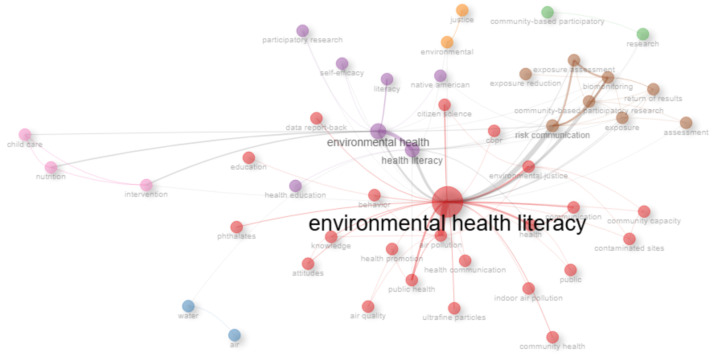
Co-occurrence networks.

Figure 12 shows three major areas of emerging research, represented by clusters of different colors. The red cluster is composed of terms such as environmental health literacy, air pollution, public health, data report-back, knowledge, attitudes, etc. The purple cluster includes terms such as environmental health, health literacy, participatory research, health education, and self-efficacy. The brown cluster encompasses terms such as risk communication, biomonitoring, and exposure assessment. In addition to these three main clusters, there are also four smaller and more isolated clusters, which include topics such as environmental justice; community-based participatory research; water and air; and child care, nutrition, and intervention.

**Figure 13:**
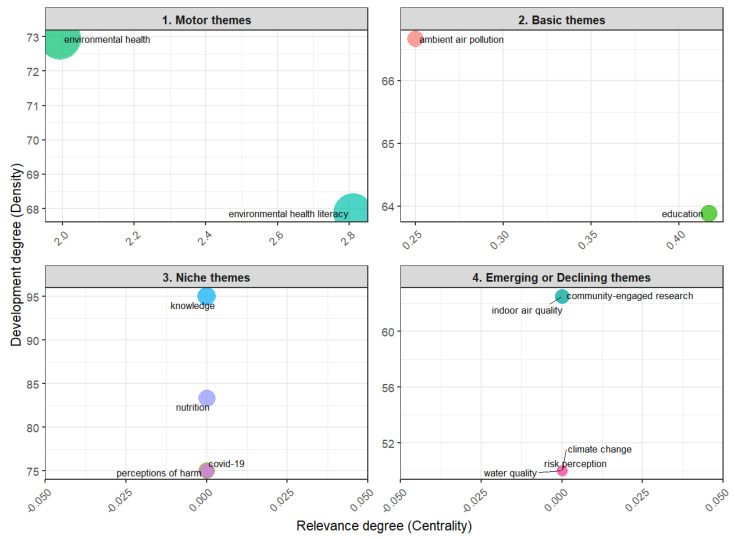
Thematic map.

The thematic map (Figure 13) categorizes the distribution of the main research themes into four groups based on their degree of development and centrality in the field. It allows for the identification of the themes that drive the research in the field, as well as the conceptual shifts [16]. The motor themes, which play an essential role in advancing research and are central to the ongoing development of the field, are in the first quadrant [21]. These themes drive the research in this field and include environmental health literacy and environmental health.

The basic themes are in the second quadrant. Despite being highly relevant, these themes are still in the early stages of development. Ambient air pollution and education are the most prominent themes of this group.

Specialized topics are in the third quadrant: niche themes. These represent more segmented areas of research with applications to specific issues. Terms such as knowledge, nutrition, COVID-19, and perceptions of harm are developed but have less centrality in the field as a whole.

Emerging or declining themes located in the fourth quadrant include topics such as community-engaged research, indoor air quality, climate change, risk perception, and water quality.

The Topic Dendrogram is a tool that shows the relationship between keywords within a search field. The data is organized hierarchically and separates the terms into distinct branches, allowing for the analysis of the thematic connections between them. The closer the terms are, the more often they are grouped together in the articles. This type of visualization helps identify the conceptual proximity between topics and the organization of areas of study within a specific field [21].

**Figure 14:**
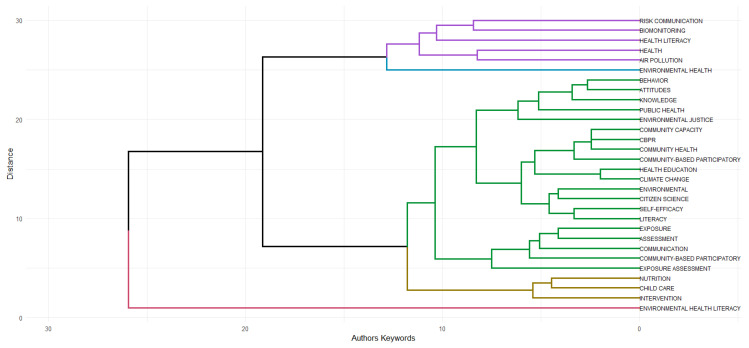
Dendrogram of Keyword Co-occurrence based on the 30 most frequent words.

In Figure 14, the green cluster represents the core of the research in environmental health literacy. Within this cluster, it is possible to observe several subgroups covering participatory methods, which involve topics such as community health, community-based participatory research, health education, and citizen science; assessment of exposure to environmental risks, which encompasses themes such as exposure, assessment, and communication; and another subgroup involving fundamental concepts of health literacy, uniting knowledge, attitudes, behaviors, public health, and environmental justice.

The purple cluster focuses on the evaluation of specific health risks. It connects risks related to air pollution with tools for measuring and communicating these risks, grouping works on topics such as air pollution, biomonitoring, risk communication, health, and health literacy.

The golden cluster is very specific, uniting topics such as nutrition, childcare, and intervention. It focuses on interventions in child health, particularly those related to nutrition. Finally, the red cluster shows that environmental health literacy is a fundamental concept connected transversely to all other topics.

#### 3.7.2. Intellectual Structure

The intellectual structure of a field of study focuses on the connections and influences established between authors, research, and sources of knowledge. It reveals the theoretical and conceptual basis underpinning the field, identifying the most influential works and highlighting how they are interconnected through citations and co-citations. This analysis enables the mapping of the main references and contributions shaping the development of the field over time [16].

**Figure 15:**
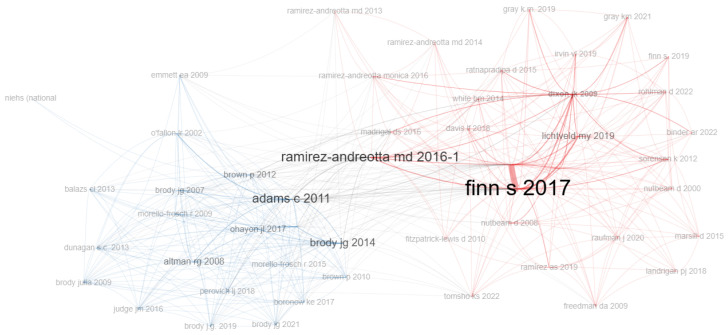
Co-citation network.

Analysis of the co-citation network (Figure 15) reveals two main clusters in the field of EHL, each representing a distinct but interconnected line of research. These clusters highlight the most influential authors in each area and illustrate the connections between different research approaches. Visualizing the network allows us to identify patterns of interaction between areas of knowledge and the most influential articles, favoring interdisciplinary collaborations and new perspectives in the study of EHL.

The red cluster is led by Finn & O’Fallon (2017) and Ramirez-Andreotta et al. (2016a) [1,2], highlighting the conceptual definition of EHL and its impact on empowering communities to face environmental risks, such as pollution and exposure to toxic substances. This group of articles focuses on the dissemination of environmental information to promote awareness and collective action, underlining the importance of education and community empowerment for public health and the environment.

The blue cluster is led by Adams et al. (2011) and Brody et al. (2014) [8,28], and focuses on the practice of reporting-back biomonitoring results and measurements of environmental exposures to study participants, paying attention to effective communication with the community and building trust. The approach commonly used is community-based participatory research, highlighting the importance of involving the community in the research process to promote environmental health literacy.

The interaction between these clusters shows that, despite their different approaches, they both share central themes, such as social justice, environmental education, and community participation. The overlap between the clusters reveals a more integrated understanding of environmental health, uniting efforts to promote a more informed and proactive society in managing its environmental risks.

#### 3.7.3. Social Structure

Social structure refers to the interactions and collaborations among authors, institutions, and countries. It helps to understand how scientific collaboration networks are formed and how they influence the production of knowledge [16].

**Figure 16:**
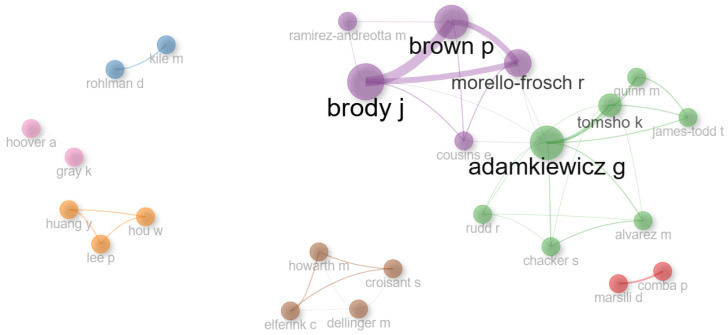
Collaboration network.

The collaboration network, Figure 16, reveals the working connections between researchers who publish on EHL. It highlights which groups are most productive, their connections with other external networks, and how these partnerships influence both the productivity and impact of research. The nodes represent the authors, while the edges indicate the intensity of the collaborations, with different weights depending on the frequency of these partnerships [21].

Ten clusters were identified, with Brody J. and Adamkiewicz K. standing out for having the largest number of collaborators. The impact of this collaborative network is to strengthen scientific production through the exchange of knowledge and the creation of more effective solutions to environmental problems. These partnerships intensify the progress of interdisciplinary studies, promoting the development of public policies and health interventions that directly benefit communities exposed to environmental risks.

## 4. Discussion

The results show a field of knowledge that is still recent but developing. Its intellectual output and impact have been growing consistently, yet not at an extremely rapid rate (Figure 2). Academic production is still sparse and emerging when compared to the broader field of Health Literacy, which had an annual output of more than 1200 articles in 2022 [29]. The youth of this research field is reflected in the fluctuating number of citations (Figure 3). The low number of citations in more recent years can be attributed to the fact that these publications have not yet had enough time to accumulate significant citations, and more time is needed to assess their impact on the scientific community. The scarcity of broader theoretical reviews is also a reflection of the recent emergence of this research field. In fact, there is a predominance of studies focusing on empirical applications.

The dominance of the US in intellectual production, followed by China (Table 4), is also observed in the broader field of Health Literacy [29]. Although it is important to note that the articles selected for this research are in English, this may underestimate production in non-English-speaking countries.

The interdisciplinary nature of the research field is reflected in the journals in which the research is published (Figure 4), bringing together the fields of environmental science and public health. Moreover, regarding the interdisciplinary nature of EHL research, the transposition of citizen science concepts and approaches deserves to be highlighted. In fact, the advancement of EHL is directly linked to the practice of reporting results in exposure studies, strengthening community engagement, and promoting behavioral changes aimed at mitigating risks [8,9,10]. The convergence of growth indicators, co-citation networks, and thematic shifts reveal that the field is moving towards community-engaged research and environmental justice frameworks. This can be seen through the use of different terms referring to communication and interaction between researchers and communities in various thematic clusters, such as data report-back, participatory research, health communication, citizen science, and community-based participatory research (Figure 12 and Figure 14).

The focus on communicating risks and establishing trusting relationships with the community through participatory research reinforces the importance of presenting research results in a clear and accessible way. This approach aims to expand people’s knowledge about risks and empower communities to make more informed decisions. However, this process faces significant barriers, such as disparities in access to information and a lack of trust in research institutions and governments [2]. In this sense, one avenue for future research concerns adjusting communication strategies to account for cultural and social barriers, utilizing dissemination channels that are accessible and relevant to local communities.

Environmental exposure monitoring projects that directly involve communities in data collection offer a way to engage populations in geographically and socially diverse regions. By integrating communities into the data collection and analysis process, interventions not only gain greater legitimacy but also become more effective and sustainable, as the proposed solutions directly reflect local needs and priorities [2,27].

Community participation is a cross-cutting theme in EHL research, which is evidenced in the co-occurrence networks in Figure 12, where this theme occurs in several clusters, and in the topic dendrogram, Figure 14. However, the under-representation of studies exploring the participation of vulnerable communities is a gap in this literature. This is further supported by the thematic map (Figure 13), which situates community-engaged research in the fourth quadrant, indicating that while it is a developing front, it still lacks the density of more established motor themes. This lack of studies reflects the complexity of integrating environmental, social, and public health issues in vulnerable regions. There are some studies focusing on vulnerable populations [30,31], but they are still scarce. Involving communities in participatory research processes is challenging in areas where access to resources is limited and where the infrastructure for data collection and analysis is inadequate. In addition, feedback on the results is often insufficient or does not effectively reach the communities. The lack of funding for these initiatives is also a significant obstacle [2,27].

Another gap is the scarcity of studies on climate-related health literacy, which is an emerging topic (Figure 13). It is worth noting that advancing research in this area could enhance public awareness of the complex relationship between health and climate change, improve the perception of health risks associated with extreme climate events, support the development of public policies and awareness-raising initiatives, empower communities, and promote behavioral changes aimed at sustainable practices and climate change adaptation. Additionally, this study identifies a disconnect between EHL and traditional EH research. Topics such as food sanitation and safety, water supply and management, environmental health impact assessment, rodent and vector control measures appear to be underrepresented in the EHL literature. This can be explained by the focus of the EHL literature on individual and community knowledge and behaviors, rather than public and state actions. This finding indicates that there is a vast field yet to be explored from a literacy perspective.

Overcoming these challenges requires a coordinated and continuous effort between policymakers, researchers, and community leaders. Partnerships between these actors are essential for creating interventions that are not only based on scientific evidence but also respect and reflect the cultural and social particularities of the most vulnerable populations. Implementing specific programs aimed at strengthening EHL in marginalized regions can be an effective strategy for reducing environmental and health disparities and promoting greater engagement and inclusion of these populations in the decision-making process [32].

When it comes to environmental justice, the collaboration and co-occurrence networks (Figure 12) suggest that this is an important topic but is still little explored in the literature, especially concerning the implementation of practical actions in communities affected by environmental risks. Although there is growing interest in addressing inequalities in environmental exposures, there is a need to transform this knowledge into public policies that guaranty equity. To do this, it is important not only to recognize disparities but also to develop effective mechanisms to redistribute resources and guaranty equitable access to environmental protection tools [9].

Finally, when analyzing the gaps identified in the areas of social justice and community participation, it is clear that more resources need to be allocated to research that integrates these themes more deeply into policy interventions. Although there are many descriptive studies on environmental disparities [10], few propose concrete solutions that can be translated into applicable public policies [19,33]. For environmental policies to be truly effective and equitable, it is essential that policymakers take into account not only scientific data but also the voices of the communities most affected, ensuring their inclusion and active participation in decision-making processes.

## 5. Conclusions

This study aimed to describe the scientific production on EHL through bibliometric indicators, identifying patterns, emerging trends, and gaps that offer opportunities for future research and development in the field. It was possible to observe that EHL has been consolidating itself as an essential interdisciplinary area that integrates public health, environmental issues, and community engagement. The bibliometric analysis, covering the period from 2012 to 2024, revealed significant growth in the volume of publications, reflecting the increase in academic interest and the urgency of empowering populations to make informed decisions in the face of environmental risks that directly affect public health and well-being.

The results point to a consistent focus on central themes such as environmental justice, environmental risks, and participatory research, highlighting the growing importance of integrating social and environmental dimensions in the promotion of public health. The analysis of the main articles and clusters reinforces the role of participatory approaches, such as the return of research results to the affected communities, highlighting their effectiveness in promoting concrete changes in public health and environmental justice. These practices reveal EHL as a powerful empowerment mechanism, allowing vulnerable populations to take greater control over the environmental challenges and socio-economic inequalities they face.

The impact of these initiatives goes beyond the local level: replicating these strategies in other countries offers an opportunity to strengthen public policies that promote EHL on a global scale. The ability to adapt these successful models to different geographical and cultural realities demonstrates the potential of EHL to tackle environmental crises and mitigate socio-environmental disparities in a more inclusive and sustainable way.

However, despite significant progress, the study also highlights important gaps between academic production and the effective implementation of public policies. The difficulty of integrating marginalized communities into research processes, coupled with the lack of resources aimed at developing participatory methodologies, still limits the practical application of the generated knowledge. Overcoming these challenges requires a coordinated effort, both in financing environmental justice initiatives and in promoting studies that prioritize community inclusion in a more assertive and effective manner.

For the future advancement of the field, it is imperative that new research explores innovative ways of communicating environmental risks, adapted to diverse audiences and local realities. These interventions need to be contextualized and culturally sensitive to ensure their effectiveness and adherence. The integration between scientific research and policy-making must be strengthened in order to transform knowledge into concrete actions, promoting more equitable and accessible environmental justice.

## Figures and Tables

**Table 1:** Bibliometric indicators and analytical purposes.

Category	Indicator & Definition	Expected Insight
Productivity & Impact	Scientific Production: Number of articles taken by authors, sources, and years	Provides measures of author productivity, sources, as well as the evolution and maturity of the field
	Lotka’s Law: Model for the frequency of author productivity.	Identifies the concentration of knowledge production among few authors.
	Citations: Number of citations taken by authors, sources, and years	Provides measures of the impact and visibility of academic output over time
	H, G, and M Indices: Metrics that balance publication quantity and citation count.	The H-index covers the relationship between the number of publications and citations of authors or sources; the G-index weighs the most cited articles, highlighting peaks of impact; and the M-index weighs production over time, allowing for evaluation of production by the author’s career stage.
Conceptual Structure	Most relevant words: Keyword occurrence	Indicates the focus of the studies in the area
	Trend Topics: Temporal analysis of keyword occurrences over time	Identifies the development of research interests
	Coupling Maps: Identifies groups of articles that share common references	Suggests the formation of subfields or research clusters
	Co-occurrence networks: Co-occurrence of words that appear together in keywords, abstracts, and titles	Allows for the identification of how certain concepts are connected
	Thematic map: Categorizes the distribution of the main research themes based on the degree of development and centrality in the field	Enables the identification of motor themes driving the field, emerging topics in early development, specialized niches, and declining trends
	Dendrogram: Hierarchical clustering of terms	Allows the visualization of the conceptual proximity and thematic grouping of keywords
Intellectual and Social Structures	Co-citation Analysis: Links documents cited together by third-party articles	Enables the identification of the most influential works and how they are interconnected
	Collaboration Analysis: Maps co-authorship between authors, institutions, and countries.	Reveals the working connections between researchers, institutions, and countries

Note: Based on Aria and Cuccurullo (2017) [16].

**Table 2:** Productivity indices for different sources.

Source	Articles	Citations	H-Index	G-Index	M-Index
*Int. J. Environ. Res. Public Health*	30	459	13	21	1.30
*Environmental Health Perspectives*	7	248	6	7	0.42
*Environmental Research*	6	75	5	6	0.55
*BMC Public Health*	5	48	3	5	0.37
*Environmental Justice*	5	21	3	4	0.21
*Environmental Health*	4	138	4	4	0.33
*Journal of Health Communication*	3	113	3	3	0.43

**Table 3:** Authors with the greatest impact on EHL.

Author	H-Index	G-Index	M-Index	Citations	Articles
Brody J	9	12	0.643	420	12
Brown P	7	10	0.500	367	10
Ramirez-Andreotta M	7	8	0.700	200	8
Morello-Frosch R	6	7	0.429	264	7
Adamkiewicz G	5	8	0.625	99	8
Marsili D	5	6	0.455	50	6
Hoover A	4	4	0.571	36	4
Rudd R	4	4	0.571	34	4
Alvarez M	3	3	0.600	24	3
Brugge D	3	4	0.333	30	4

**Table 4:** Scientific production and total citations by country.

Countries	Scientific Production	Total Citations
USA	441	1442
China	50	41
Italy	35	53
Australia	24	51
Thailand	14	14
United Kingdom	14	5
Canada	10	19
Portugal	9	0
Serbia	8	0
South Korea	8	14

## Data Availability

Research data can be provided under reasonable request.

## References

[1] Finn S., O’Fallon L. (2017). The emergence of environmental health literacy—From its roots to its future potential. Environ. Health Perspect..

[2] Ramirez-Andreotta M., Brody J., Lothrop N., Loh M., Beamer P., Brown P. (2016). Improving environmental health literacy and justice through environmental exposure results communication. Int. J. Environ. Res. Public Health.

[3] Gray K. (2018). From content knowledge to community change: A review of representations of environmental health literacy. Int. J. Environ. Res. Public Health.

[4] Lichtveld M.Y., Covert H.H., Sherman M., Shankar A., Wickliffe J.K., Alcala C.S. (2019). Advancing environmental health literacy: Validated scales of general environmental health and environmental media-specific knowledge, attitudes and behaviors. Int. J. Environ. Res. Public Health.

[5] Ratnapradipa D., Getz T.D., Zarcadoolas C., Panzara A.D., Esposito V., Wodika A.B., Caron C., Migliore B., Quilliam D.N. (2010). Environmental Health Risk Communication: Assessing Levels of Fish Consumption Literacy Among Selected Southeast Asians. Appl. Environ. Educ. Commun..

[6] Irvin V.L., Rohlman D., Vaughan A., Amantia R., Berlin C., Kile M.L. (2019). Development and Validation of an Environmental Health Literacy Assessment Screening Tool for Domestic Well Owners: The Water Environmental Literacy Level Scale (WELLS). Int. J. Environ. Res. Public Health.

[7] Matlack M., Covert H., Shankar A., Zijlmans W., Abdoel Wahid F., Hindori-Mohangoo A., Lichtveld M. (2023). Development of a Pilot Literacy Scale to Assess Knowledge, Attitudes, and Behaviors towards Climate Change and Infectious Disease Dynamics in Suriname. Int. J. Environ. Res. Public Health.

[8] Brody J., Dunagan S., Morello-Frosch R., Brown P., Patton S., Rudel R. (2014). Reporting individual results for biomonitoring and environmental exposures: Lessons learned from environmental communication case studies. Environ. Health.

[9] Eggers M., Doyle J., Lefthand M., Young S., Moore-Nall A., Kindness L., Medicine R., Ford T., Dietrich E., Parker A. (2018). Community Engaged Cumulative Risk Assessment of Exposure to Inorganic Well Water Contaminants, Crow Reservation, Montana. Int. J. Environ. Res. Public Health.

[10] Brown P., Brody J.G., Morello-Frosch R., Tovar J., Zota A.R., Rudel R.A. (2012). Measuring the Success of Community Science: The Northern California Household Exposure Study. Environ. Health Perspect..

[11] Mansour A., Gatto M.R., Rowbotham S., Bowen K., Bentley R. (2025). Climate-related healthy housing risk factors: A scoping review of citizen science approaches. Environ. Sci. Policy.

[12] Villa G., Rosa D., Marcomini I., Poliani A., Spena P.R., Buccione R., Manara D.F., Fedeli M. (2025). Community-driven research: Exploring the potential of citizen science in nursing. Int. J. Nurs. Stud..

[13] Stanifer S., Goodman Hoover A., Rademacher K., Rayens M.K., Haneberg W., Hahn E.J. (2022). Citizen Science Approach to Home Radon Testing, Environmental Health Literacy and Efficacy. Citiz. Sci. Theory Pract..

[14] Sandhaus S., Kaufmann D., Ramirez-Andreotta M. (2019). Public participation, trust and data sharing: Gardens as hubs for citizen science and environmental health literacy efforts. Int. J. Sci. Educ. Part Commun. Public Engagem..

[15] Mayntz R. (2001). El Estado y la sociedad civil en la gobernanza moderna. Rev. Del CLAD Reforma Y Democr..

[16] Aria M., Cuccurullo C. (2017). bibliometrix: An R-tool for comprehensive science mapping analysis. J. Inf..

[17] Page M.J., McKenzie J.E., Bossuyt P.M., Boutron I., Hoffmann T.C., Mulrow C.D., Shamseer L., Tetzlaff J.M., Akl E.A., Brennan S.E. (2021). The PRISMA 2020 statement: An updated guideline for reporting systematic reviews. BMJ.

[18] Ramos I.N., He Q., Ramos K.S. (2012). Improvements in Environmental Health Literacy along the Texas-Mexico Border Following Community-wide Health Education. Environ. Justice.

[19] Ohayon J., Cousins E., Brown P., Morello-Frosch R., Brody J. (2017). Researcher and institutional review board perspectives on the benefits and challenges of reporting back biomonitoring and environmental exposure results. Environ. Res..

[20] Claudio L., Gilmore J., Roy M. (2018). Communicating environmental exposure results and health information in a community-based participatory research study. BMC Public Health.

[21] Silva C.L., Sgarbossa M., Grzybovski D., Mozzato A.R. (2022). Manual práTico para Estudos Bibliométricos Com o Uso do Biblioshiny.

[22] Alvarado R. (2002). A lei de Lotka na bibliometria brasileira. Lotka’s Law in Brazilian bibliometrics. Ciênc. Inform..

[23] Ramírez A.S., Ramondt S., Bogart K., Perez-Zuniga R. (2019). Public awareness of air pollution and health threats: Challenges and opportunities for communication strategies to improve environmental health literacy. J. Health Commun..

[24] Dodson R.E., Boronow K.E., Susmann H., Udesky J.O., Rodgers K.M., Weller D., Woudneh M., Brody J.G., Rudel R.A. (2020). Consumer behavior and exposure to parabens, bisphenols, triclosan, dichlorophenols, and benzophenone-3: Results from a crowdsourced biomonitoring study. Int. J. Hyg. Environ. Health.

[25] Ramirez-Andreotta M., Brody J.G., Lothrop N., Loh M., Beamer P.I., Brown P. (2016). Reporting back environmental exposure data and free choice learning. Environ. Health.

[26] Perovich L.J., Ohayon J.L., Cousins E.M., Morello-Frosch R., Brown P., Adamkiewicz G., Brody J.G. (2018). Reporting to parents on children’s exposures to asthma triggers in low-income and public housing, an interview-based case study of ethics, environmental literacy, individual action, and public health benefits. Environ. Health.

[27] Madrigal D., Minkler M., Parra K., Mundo C., Cardenas Z. (2016). Improving Latino youths’ environmental health literacy and leadership skills through participatory research on chemical exposures in cosmetics: The HERMOSA study. Int. Q. Community Health Educ..

[28] Adams C., Brown P., Morello-Frosch R., Brody J.G., Rudel R., Zota A., Dunagan S., Tovar J., Patton S. (2011). Disentangling the Exposure Experience: The Roles of Community Context and Report-Back of Environmental Exposure Data. J. Health Soc. Behav..

[29] Tabak B.M., Froner M.B., Corrêa R., Silva T.C. (2023). The Intersection of Health Literacy and Public Health: A Machine Learning-Enhanced Bibliometric Investigation. Int. J. Environ. Res. Public Health.

[30] Buta B.O., Manchineri W.S.C.S., Froner M.B., Ecija M.B., Cardoso D.H.R., Tabak B.M. (2025). Environmental Health Literacy of Brazilian Indigenous People. Int. J. Environ. Res. Public Health.

[31] Davis L.F., Ramirez-Andreotta M.D., McLain J.E.T., Kilungo A., Abrell L., Buxner S. (2018). Increasing Environmental Health Literacy through Contextual Learning in Communities at Risk. Int. J. Environ. Res. Public Health.

[32] Sisson S., Salvatore A., Hildebrand D. (2019). Interventions to promote healthy environments in family child care homes in Oklahoma—Happy Healthy Homes: Study protocol for a randomized controlled trial. Trials.

[33] Buta B.O., Froner M.B., Tabak B.M. (2025). Scale validation and prediction of environmental health literacy in Brazil. Sci. Rep..

